# Short-term changes related to autotetraploidy in essential oil composition of *Eucalyptus benthamii* Maiden & Cambage and its applications in different bioassays

**DOI:** 10.1038/s41598-021-03916-2

**Published:** 2021-12-23

**Authors:** Alex Junior da Silva, Wellington Ronildo Clarindo, Guilherme Ferreira Simiqueli, Milene Miranda Praça-Fontes, Luiza Alves Mendes, Gustavo Ferreira Martins, Aluízio Borém

**Affiliations:** 1 Departament of Agronomy, Federal University of Viçosa, Viçosa, MG ZIP 36570-900 Brazil; 2 Departament of General Biology, Federal University of Viçosa, Viçosa, MG ZIP 36570-900 Brazil; 3 Departament of Biology, Federal University of Espírito Santo, Alegre, ES ZIP 29500-000 Brazil; 4 Departament of Chemistry, Federal University of Viçosa, Viçosa, MG ZIP 36570-900 Brazil

**Keywords:** Secondary metabolism, Plant breeding, Forestry

## Abstract

Some forest trees have been polyploidized to improve their traits and to supply new germplasms for breeding programs. As trees have a long juvenile stage, the early characterization of the chromosome set doubling effects is crucial for previous selection. Thus, we aimed to characterize the chemical variability of essential oils from diploid and autotetraploid germplasms (autotetraploid A and B) of *Eucalyptus benthamii*, as well as to evaluate their larvicidal and allelopathic effects. Autotetraploid A showed a higher essential oil yield than diploid and autotetraploid B, which did not differ quantitatively. Aromadendrene, viridiflorol and α-pinene were the major compounds in the diploid essential oil. In contrast, compounds were present in autotetraploids, such as 1,8-cineole, limonene, α-terpineol, and α-terpinyl-acetate. Essential oils from the diploid at 50–200 ppm were twice as larvicidal than those from autotetraploids against *Aedes aegypti* larvae. Considering the phytotoxicity bioassays using *Lactuca sativa*, essential oils from both ploidy levels affected root growth. Moreover, the essential oils inhibited shoot growth at all concentrations tested (187.5; 375; 750; 1500; and 3000 ppm). Autotetraploid A and B had the same effect on shoot growth as glyphosate. The essential oils had no cytogenotoxic effect on root meristematic cells of *L. sativa,* whereas phytotoxic potential was identified mainly in shoot growth. This work demonstrated a dramatic change in secondary metabolism (terpene composition) related to an increase in the ploidy level in *Eucalyptus* germplasms. In addition, we report the novelty of the chemical composition of essential oils among germplasms and their potential use as larvicidal and post-emergence weed control agents.

## Introduction

*Eucalyptus* is an important genus among the cultivated woody angiosperms, and it includes more than 700 species of trees and shrubs^1^. *Eucalyptus* belongs to the Myrtaceae Family, which is prolific in species that produce essential oils (EOs)^2^. From more than 300 different EOs of commercial value, at least 20 are found in the genera *Eucalyptus* and *Corymbia*, which possess species characterized by high EO production^3^.

Exploration of *Eucalyptus* EOs dates from the nineteenth century in Australia^4^. Since then, advances in silviculture have been optimized to maximize EO production, such as selection and crossing involving superior genotypes^5^. Breeders were able to select important phenotypic features to improve EO yield and tree adaptation to different environments in some species, such as *Corymbia citriodora* (Hook.) K.D. Hill & L.A.S. Johnson*, Eucalyptus camaldulensis* Dehnh.*, Eucalyptus radiata* D. C.*, Eucalyptus globulus* Labill., and *Eucalyptus staigeriana* F. Muell^6^. Phytochemically, *Eucalyptus* EOs can have citronellal, citronellol, 1,8-cineole, α-pinene, α-terpineol, limonene, geranyl acetate and linalool, among other major compounds^7^. The chemical components, as well as the yield of EOs, may vary according to the environment. For example, *E. camaldulensis* from Argentina contains 1,8-cineole, p-cymene, β-phellandrene, and 0.38% EO yield, while in Brazil, the same species has 1,8-cineole, limonene, γ-terpinene, α-pinene, and 0.63% yield^8,9^. Despite the characterization of EO from *E. benthamii* leaves^10^, these trees are mostly planted for the production of cellulose, timber, or bioenergy. For example, leaves are left in the field as litter for biomass decomposition and have not yet been explored for EO extraction.

Aiming for germplasm diversity, synthetic autotetraploid and allotetraploid *Eucalyptus* have been produced to be incorporated in breeding programs and in the field^11,12^. As the *Eucalyptus* nuclear genome potentially has 113 genes related to terpene synthesis^13^, chromosome set doubling (CSD) procedures increase the gene copy and, consequently, increase the phenotypic diversity of EO production and composition^14,15^. Therefore, the gene copy increases alleles per locus, generating more overdominance than in diploid counterparts^16^. The response for this effect covers the “omics” (genomic, epigenomic, transcriptomic, and metabolomic), resulting in different yield and composition of secondary metabolites, such as EO^17^. In spite of CSD could enhance EO production, a careful screening and selection still needed among the autopolyploid or allopolyploid germplasms, or both, based on chemical profiling^15,18,19^.

EO synthesis plays a central role in plants, providing defense against biotic and abiotic stresses^20^. Due to their chemical variability and toxicity, EOs are biomolecules extensively used as pesticides to control insect pests, plant diseases, and weeds^7,8,10,21^. Neotropical countries, such as Brazil, have suffered from dengue, zika, yellow fever, and chikungunya epidemics, transmitted by *Aedes aegypti* L.^27^. To control *A. aegypti*, health agencies frequently use pyrethroids as insecticides, which have larvicidal effect at concentrations as low as 1 ppm. However, this control method can promote insect resistance and affect non-target organisms^23,24^. Likewise, synthetic herbicides are applied indiscriminately and has caused problems to the environment and other organisms. Thereby, compounds of natural origin are considered promising in the obtention of novel pesticides that cause less impact^25,26^.

In the present study, we described and compared the chemical composition of foliar EOs of one diploid and two synthetic autotetraploid *E. benthamii* germplasms. Additionally, we tested the larvicidal and allelopathic effects of these EOs. We used *Aedes aegypti* and *Lactuca sativa* L. in toxicity bioassays. Allelopathic effects of EOs were described by reducing seed germination, rate germination, as well as shoot and root growth of plantlets. The identification of aneugenic and clastogenic effects (such as chromatin condensation, micronuclei, and chromosome loss and alterations) was also used as a parameter to determine the toxicity of EOs. Moreover, flow cytometry analysis was used to verify the effect of EOs on cell proliferation.

## Materials and methods

### Plant material and ploidy level stability

Seeds of *E. benthamii* from open-pollinated crosses were germinated, and a diploid germplasm (2n = 2 ×  = 22 chromosomes) was obtained and multiplied. The seed lot was licensed and provided by Klabin S.A. Solid autotetraploid plantlets (2C = 1.22 pg, 2n = 4 ×  = 44 chromosomes) were generated from two diploid plantlets submitted to the CSD procedure, using 1,5 mM of colchicine for 36 h of treatment exposure^12^. Considering that *Eucalyptus* is predominantly allogamous and the germplasms were generated from two distinct seeds of *E. benthamii,* the autotetraploids were denominated here as autotetraploid A and autotetraploid B. Diploid and autotetraploids were vegetative propagated and developed during 2016 and 2017 at the Laboratório de Citogenética e Citometria (Universidade Federal de Viçosa, MG, Brazil). The multiplication media used was 4.3 g L^−1^ MS basal salts (Sigma®) supplemented with 0.5 mg L^−1^ 6-benzylaminopurine (Sigma®) and 0.05 mg L^ − 1^ α-naphthaleneacetic (Sigma®). The ploidy level stability was checked by flow cytometry during in vitro propagation^12^.

After three months of in vitro multiplication, the plantlets were acclimatized and transferred to the Klabin S.A. Experimental Research Station, Paraná, Brazil (24°13′31″S, 50°32′44″O). The trees were cultivated for 1.5 years and spaced 3 × 2 m, until they reached 5-m high and 12.5-m circumference at breast height. In the field, a new ploidy assessment was performed to check the DNA ploidy level stability. Leaves from nine trees (three from each germplasm) were collected during Autumn (April/May 2019), in three different portions along the longitudinal axis: basal, medium, and top of the crown. For harvesting, trees were cut and processed within 30 min each, 500 g of leaves were packed in kraft paper for each sample. After 24 h, the leaves were air-dried for seven days at 25 ± 1 °C, and weighed in an analytical balance to obtain the leaves’ dry mass. All samples were stored at − 20 °C until use^27^. The use of the plant material in this study is in accordance with Klabin S.A., national and international guidelines.

### EO extraction and yield

Leaves (50 g) from each sample were added to a round-bottom distillation flask (1 L) containing 600 mL of dH_2_O. The flasks were attached to a Clevenger apparatus and a condenser. Hydrodistillation was performed for 3 h, and the vapor of oils and water was condensed and collected into a 125 mL Erlenmeyer flask, as recommended by the Brazilian Phamacopoeia for volatile oils^7,28^. The hydrolate was subjected to liquid–liquid extraction using dichloromethane (3 × 10 mL). The organic phase was dried with anhydrous sodium sulfate, filtered, and concentrated under reduced pressure in a rotatory evaporator. The obtained EO was transferred to Eppendorff® microtubes and protected from the light with an aluminum foil, and stored at − 20 °C for 5 days. The EO yield was measured considering the dried mass of the leaves divided by the extracted EO mass in m m^−1^^7^.Each sample from every germplasm was ranked according to EO yield (%) and denominated as 1 (lower yield), 2 (intermediate yield), and 3 (higher yield). The yield data were subjected to ANOVA and the Scott-Knott test at 5%, and a correlation analysis was performed between yield (%) and composition (%).

### Chromatographic profile of EO

The extracted EO of each sample was analyzed via gas chromatography with a flame ionization detector (GC-FID, Shimadzu GC-2010 Plus) and gas chromatography coupled to mass spectrometry (GC–MS, Shimadzu GCMS-QP2010 SE). EOs were removed from the vials in 1 μL of a solution consisting of 3% EOs dissolved in n-hexane. The analysis was conducted following the equipment conditions: Helium (He) as carrier gas in both detectors, with flow and linear speed of 2.80 mL min^−1^ and 50.8 cm s^−1^ (GC-FID), and 1.98 mL min^−1^ and 50.9 cm s^−1^ (GC–MS), respectively; 220 °C injector temperature in split ratio of 1:30; fused silica capillary column (30 m × 0.25 mm); stationary phase Rtx®-5MS (0.25 µm film thickness); oven program with initial temperature of 40 °C for 3 min followed by graduated increments of 3 °C min^−1^ up to 180 °C, which was maintained for 10 min for a total analysis time of 59.67 min; and 240 °C FID and 200 °C MS detector temperatures^27,29^. GC–MS analysis was performed in electronic impact equipment with 70 eV impact energy, 1000 scan speed, 0.50 fragment s^−1^ scanning interval, and fragment detection from 29 to 400 (m/z). GC-FID analysis was performed using an H_2_ flame and atmospheric air at 300 °C. Flow speeds used for H_2_ and air were 40 mL min^−1^ and 400 mL min^−1^, respectively.

The identification of the EO components was performed by comparing the obtained mass spectra to those available in the spectral library database (Wiley 7, NIST 05, and NIST 05s) and the retention index (RI). To calculate the RI, a sample of saturated alkanes C_7_–C_40_ (Supelco, USA) and the adjusted retention time of each compound were employed as obtained by GC-FID. Subsequently, the values calculated for each compound were compared to those reported in the literature^30^. The percentage of each compound in the EO was calculated as the ratio between the integral area of the peaks and the total area of all sample constituents, as obtained from the GC-FID analysis. Compounds with a relative area > 1% were identified, and those > 5% were considered to be predominant.

### Larvicidal activity

For larvicidal activity, three EOs (1, 2, and 3) from diploids, autotetraploids A, and autotetraploid B were selected based on lower, intermediate, and higher yields (%). The phytotoxicity and cytotoxicity bioassays were performed with two EOs (lower and higher yields) of the diploid autotetraploids A and B.

In order to verify the larvicidal activity of the EOs from diploids and autotetraploids *E. benthamii*, fourth-instar larvae of *A. aegypti* (PPCampos strain, Campos Goytacazes, RJ) were used. The larvae were obtained from a colony maintained at the Universidade Federal de Viçosa. The eggs were incubated in trays with dechlorinated (tap) water, kept under controlled conditions (26 ± 2 °C, 12 h L:D photoperiod), and fed with turtle food (Reptolife®, Alcon,Camboriú, SC, Brazil) until the L4 stage^25^. EO solutions were prepared with 1% dimethyl sulfoxide (DMSO, v v^−1^) at concentrations of 50, 75, 100, 125, 150, 175, and 200 ppm^25^. The larvae were collected with a Pasteur pipette and distributed into glass vials containing 30 mL of the EO solutions. Control solutions were prepared with 1% DMSO, as a negative control, and deltamethrin 0.07 ppm (Decis 25 EC, 25 g L^−1^, emulsifiable concentrate, Bayer S.A., Germany), as a positive control. The assay was performed with five repetitions for each treatment with 25 larvae each (125 larvae per treatment), without solution replacement; after 24 h, *A. aegypti* mortality was assessed. The larvae were considered dead when no response to stimuli was found (direct contact with a Pasteur pipette). Relationships between different EOs and their concentrations were evaluated with a regression analysis (9 oils × 7 concentrations). A generalized linear model was used, with a Poisson distribution of residual effect and logarithmic ligation function. The following factors EOs, concentration, and its interaction were evaluated with deviance analysis at 5% probability of type 1 error. Confidence intervals of 95% of probability were built for each concentration of EO with the Effects package^31^. All analyses were performed with R software^32^.

### Phytotoxicity bioassay

A phytotoxicity bioassay was performed using EO concentrations tested at 187.5, 375, 750, 1500, and 3000 ppm^33,34^. The statistical design was completely randomized with 5 replications for each treatment. Each experimental unit consisted of a Petri dish (10 cm × 2 cm) containing 25 seeds of *L. sativa* ‘Itapuã Super’ (Isla S.A., Brazil), which were placed on a piece of filter paper. EOs were dissolved in dichloromethane and applied using a sterile Pasteur pipette. The evaluated variables after exposure to EOs were as follows: (a) germination rate (GR, %) and root growth (RG, mm) after 48 h of exposure; (b) germination speed index (GSI), calculated every 8 h during 48 h, using the formula (N1 ^.^ 1) + (N2 − N1) ^.^ 1/2 + (N3 − N2) ^.^ 1/3 + … (Ny − (Ny − 1)) ^.^ 1/y (where Ny: number of germinated seeds in a given period; Y: total number of evaluations); and (c) shoot growth (SG, mm), determined after 120 h of exposure. The phytotoxicity bioassay was analyzed through a multiple linear regression with dummy variables, comparing each variable with the following factors: negative controls (water and dichloromethane), positive control (glyphosate), and EOs (diploid and autotetraploids A and B) as qualitative independent variables, and concentrations of EOs as quantitative independent variables. ANOVA for regression was performed at 5% probability and 95% confidence intervals were built for each concentration of EO with Effects package^31^. The data were analyzed using R software^32^.

### Cytogenotoxicity bioassays

After 48 h in the phytotoxicity bioassay, 10 roots of *L. sativa* with the same, intermediate, and highest RG compared with controls, were fixed three times in fresh cold methanol:acid acetic acid (3:1), and stored overnight at − 20 °C. The roots were fixed again and stored at − 20 °C overnight in 70% ethanol. The influence of EOs on cell proliferation was verified by flow cytometry, comparing the number of G_2_/M cells in root meristems of each treatment in relation to the positive and negative controls (water, dichloromethane, and glyphosate). Flow cytometry analysis was conducted with four repetitions being that roots of five plants of each treatment were considered an experimental unit. Nuclei were isolated by macerating roots with a pestle five times in 700 µL Otto-I lysis buffer^35^. After 3 min, the suspensions were filtered through a 30-µm nylon mesh (Partec®, Germany) and centrifuged at 1100 rpm for 5 min to separate the supernatant to be discarded. The pellets were eluted in 100 µL Otto-I lysis buffer for 10 min, and the nuclei suspensions were stained in 300 µL Otto-II solution^36^ supplemented with 75 µM propidium iodide and immediately filtered through a 20-µm nylon mesh (Partec®). The homogenates were incubated in the dark for 20 min at 25 ± 1 °C. The nuclei suspensions were analyzed in a BD Accuri C6 (Accuri cytometers, Belgium) equipped with a laser source to detect emissions at FL3 (> 670 nm). The histograms were analyzed using the BD CSampler™ software.

Additionally, clastogenic and aneugenic effects were evaluated from slides prepared from the same treatments used for flow cytometry analysis. Five previously fixed roots of *L. sativa* per treatment were used. Each root was washed in dH_2_O for 10 min, and subjected to hydrolysis in 5 N hydrochloric acid at 25 °C for 18 min. Each root meristem was placed on a glass slide with one drop of 2% orcein acetic solution, and covered with a coverslip. Then, pressure was applied on the coverslip to macerate the root meristem^33^. The following variables were analyzed: (a) % of chromosomal alterations (AC), (c-metaphasis + anaphase bridges + chromosome fragments) / total number observed cells (10.000 cells); and (b) micronuclei occurrence.

## Results

### EO extraction, profiling, and germplasm characterization

Considering the stratification of leaves along the longitudinal axis, the EO yield (%) and chemical composition were the same. However, the yield and chemical composition of the EOs differed among the diploid and autotetraploid germplasms. Autotetraploid A showed higher yields (1.71–7.21%; w w^−1^) compared to the diploids (1.08–2.3%; w w^−1^) and autotetraploid B (0.65–1.98% w w^−1^). Despite the ploidy level difference, the EO yield was the same between the diploids and the autotetraploid B (Tables 1 and S1).

**Table 1:** Yield (%) and chemical composition (%) of the *E. benthamii* EOs. Diploid and Autototetraploids A and B germplasms were vegetative propagated. Identification 1, 2 and 3 refers to yield (lower, intermediate and higher, respectively).

Germplasm	Ploidy	Yield	Aromadendrene	1,8-cineole	Limonene	Viridiflorol	α-pinene	α-terpineol	α-terpinyl acetate
Diploid 1	2x	1.16	16.92	–	–	19.78	63.3	–	–
Diploid 2	2x	1.75	22.1	–	–	18.74	59.16	–	–
Diploid 3	2x	2.30	22.61	–	–	21.83	55.56	–	–
Autotetraploid A1	4x	1.79	–	58.72	–	–	29.21	12.07	–
Autotetraploid A2	4x	4.31	–	54.32	–	–	33.17	12.51	–
Autotetraploid A3	4x	7.21	–	55.93	–	–	31.14	12.93	–
Autotetraploid B1	4x	0.64	–	52.10	5.14	–	34.13	–	8.63
Autotetraploid B2	4x	1.73	–	51.19	5.47	–	35.49	–	7.85
Autotetraploid B3	4x	1.98	–	49.78	5.10	–	31.45	–	8.62

Gas chromatography showed that the diploids EOs presented α-pinene (60.43%), viridiflorol (19.93%), and aromadendrene (19.64%); both autotetraploids A and B EOs presented 1,8-cineole (54.76%) and α-pinene (31.98%) as the major compounds. The compounds aromadendrene and viridiflorol present in diploid EOs were not identified in autotetraploid EOs. In addition, autotetraploid EOs presented different compounds: autotetraploids A, α-terpineol (13.21%) and autotetraploids B, α-terpinyl-acetate (8.46%) and limonene (4.75%). A single sample from autotetraploid B also exhibited trans-β-ocimene (0.56%). The quantitative variation (%) among aromadendrene, viridiflorol, and 1,8-cineole had a positive correlation with yield (%) for each germplasm (0.62, 0.35, and 0.23, respectively) (Tables 2 and S2).

**Table 2:** Chemical identification of the compounds present in diploid and autotetraploid *E. benthamii* germplasms.

Germplasm	Ploidy	Compound	Mean (%)	Yield (%)	r (compound/yield)	n	t calc	t tab	Significance at 5%
Diploid/ A/ B	2x/4x/4x	α-Pinene	–	–	− 0.2550407	27	− 1.319	0.9605	ns
A/B	4x/4x	1,8-Cineole	–	–	0.2345942	18	0.9653	0.9607	*
Diploid	2x	Aromadendrene	19.63 ± 2.38	1.56	0.6242518	9	2.1141	0.9615	*
		Viridiflorol	19.94 ± 1.50	1.56	0.3518146	9	0.9944	0.9615	*
A	4x	α-Terpineol	12.7 ± 0.70	3.05	0.1519108	9	0.4066	0.9615	ns
B	4x	Limonene	4.75 ± 1.79	1.23	0.1018077	8	0.2507	0.9617	ns
		Trans-β-ocimene	0.56 ± 1.68	1.23	–	1	–	–	–
		α-Terpinyl acetate	8.42 ± 0.57	1.23	− 0.1324388	9	− 0.354	0.9615	ns

### Larvicidal bioassay

**Figure 1:**
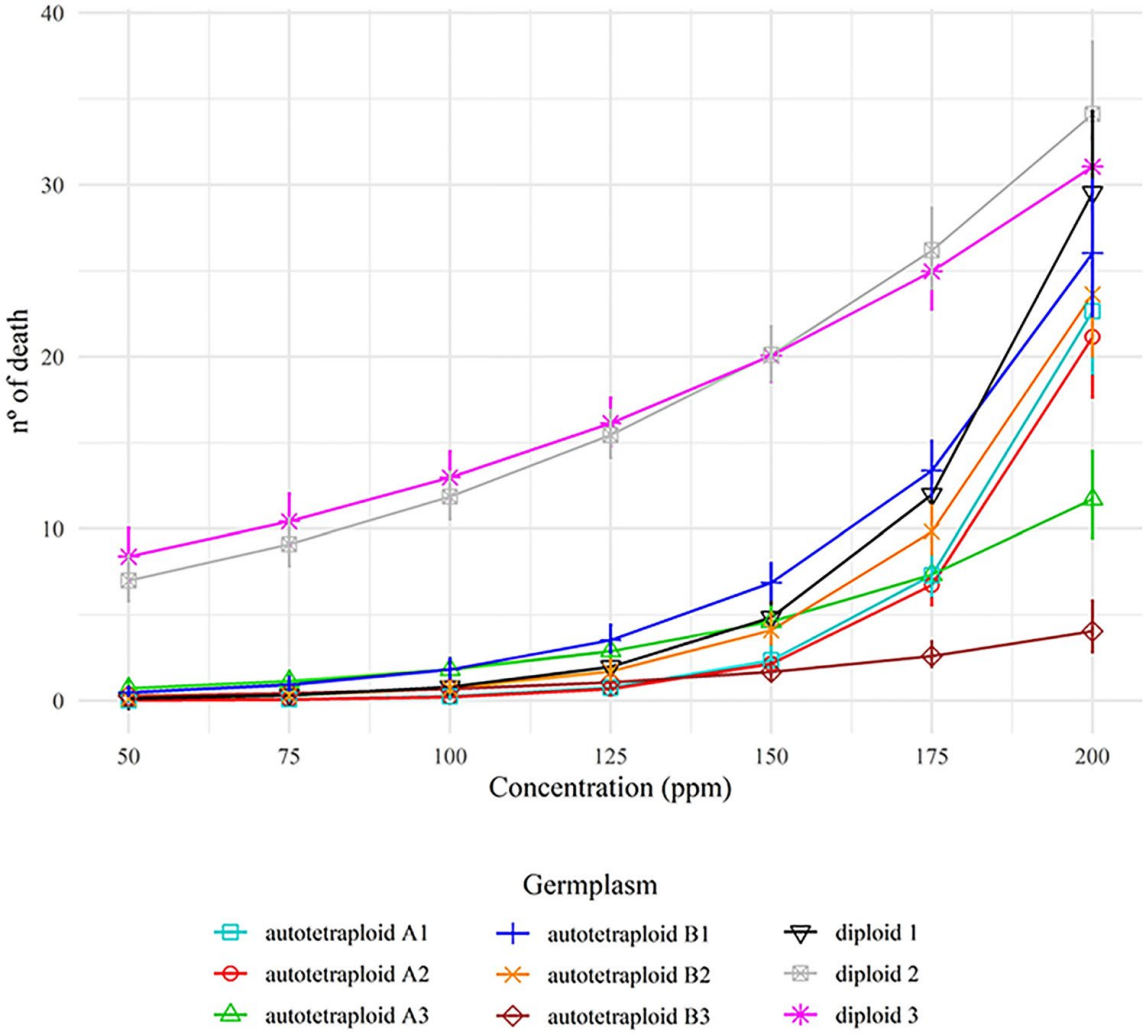
EOs from *E. benthamii* diploid and autotetraploid germplasms tested in Larvicidal bioassay. Number of dead *A. aegypti* larvae after 24 h of exposition at different concentrations of EOs. Positive control: Deltamethrin; negative control: DMSO. Germplasm: diploid 1, 2 and 3 (lower, intermediate and higher yield, respectively); autotetraploid A1, A2 and A3 (lower, intermediate and higher yield, respectively); autotetraploid B1, B2 and B3 (lower, intermediate and higher yield, respectively). The bars are 95% confidence intervals. Overlapped confidence intervals indicate that statistical difference was not detected. The number of deaths over 25 larvae estimated by the generalized linear model, suggest that 175 and 200 ppm EOs are enough to promote high mortality.

Diploid EOs ranging from 50 to 200 ppm showed larvicidal activity. EOs from diploid 2 and 3 (intermediate and higher yields) promoted 50% of larval death at 100 ppm and 80% at 150 ppm. EOs from diploid 2 and 3 showed 5.18% and 5.69% more aromadendrene, and 4.14% and 7.74% less α-pinene than diploid 1 EOs. Diploid 1 (lower yield) and autotetraploids A and B’ EOs promoted 50% of deaths only at 200 ppm (Fig. 1 and Table S3). The positive control solution with Deltamethrin caused 100% mortality; thus, treatments with 25 dead larvae had the same effect as the positive control. The negative control (DMSO) did not promote any deaths, indicating that DMSO, used as a solvent, had no relation to the larvicidal effect of EOs.

### Phytotoxicity bioassay

**Figure 2:**
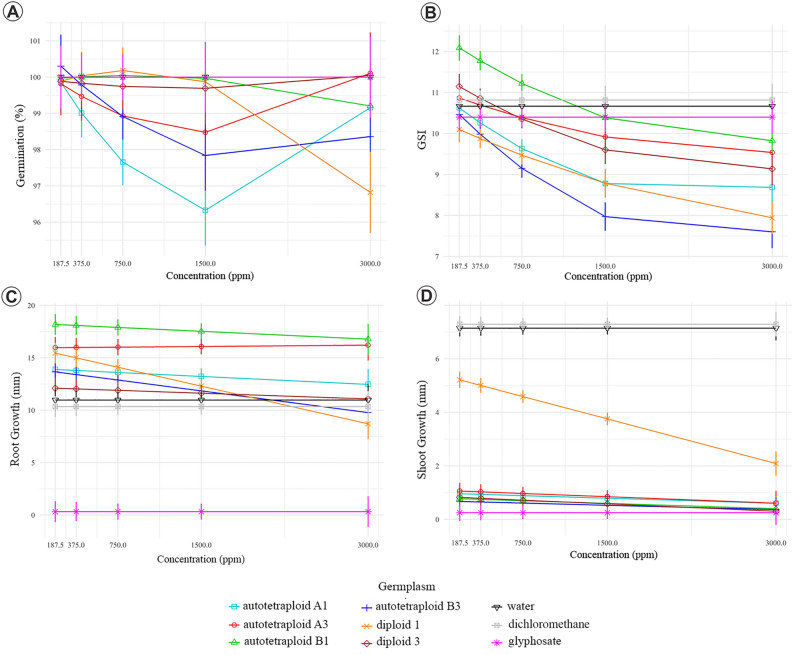
EOs from *E. benthamii* diploid and autotetraploid germplasms tested in hytotoxicity bioassays. (**a**) GR (%) of *L. sativa* seeds after 48 h of exposition; (**b**) GSI of *L. sativa* seeds after 48 h of exposition; (**c**) RG (mm) of *L. sativa* seeds after 48 h of exposition; (**d**) SG (mm) of *L. sativa* seeds after 48 h of exposition; Positive control: glyphosate; negative control: water and dichloromethane. Germplasm: diploid 1 and 3 (lower and higher yield); autotetraploid A1 and A3 (lower and higher yield); autotetraploid B1 and B3 (lower and higher yield). The bars are 95% confidence intervals. Overlapped confidence intervals indicates that statistical difference was not detected.

Most EOs from *E. benthamii* diploid and autotetraploids did not significantly reduce the GR of *L. sativa* seeds. Despite autotetraploid A1 at 750 and 1500 ppm (means = 97.65% and 96.32%, respectively), autotetraploid B3 at 1500 ppm (mean = 97.83%), and diploid 1 at 3000 ppm (mean = 96.82%) were statistically different from the positive and negative controls (mean = 100%), GR caused by these EOs was still considered high for this bioassay (Fig. 2, a). Autotetraploids A1, B3, and diploid 1 (from 375 ppm to 3000 ppm) oils also promoted a reduction in GSI (Fig. 2, b) when compared to the water control (GSI = 10.66). Seeds treated with autotetraploid B3 and diploid 1 (3,000 ppm) reached GSI = 7.6 and 7.94, respectively.

EOs from autotetraploid germplasms A1, A3, and B1 promoted RG at all concentrations. Autotetraploid B1 promoted higher RG (18.18 mm) than the water control (RG = 10.97). Seeds treated with diploid 3 and autotetraploid B3 EOs did not differ from the controls, while diploid 1 caused a reduction in RG from 187.5 ppm (15.45 mm) and to started inhibition at 3,000 ppm (8.7 mm) (Fig. 2c). However, EOs had an evident effect on post-emergence of *L. sativa* plantlets, causing a reduction of up to 6.2 mm in the SG (Fig. 2d). Autotetraploids B1 and B3 and diploid 3 EOs had the same effect as glyphosate herbicide (SG = 0.25 mm). Autotetraploid A1 and A3 EOs also reduced SG as well as diploid 1, which it was gradually (Fig. 2d).

### Cytogenotoxicity bioassay

**Figure 3:**
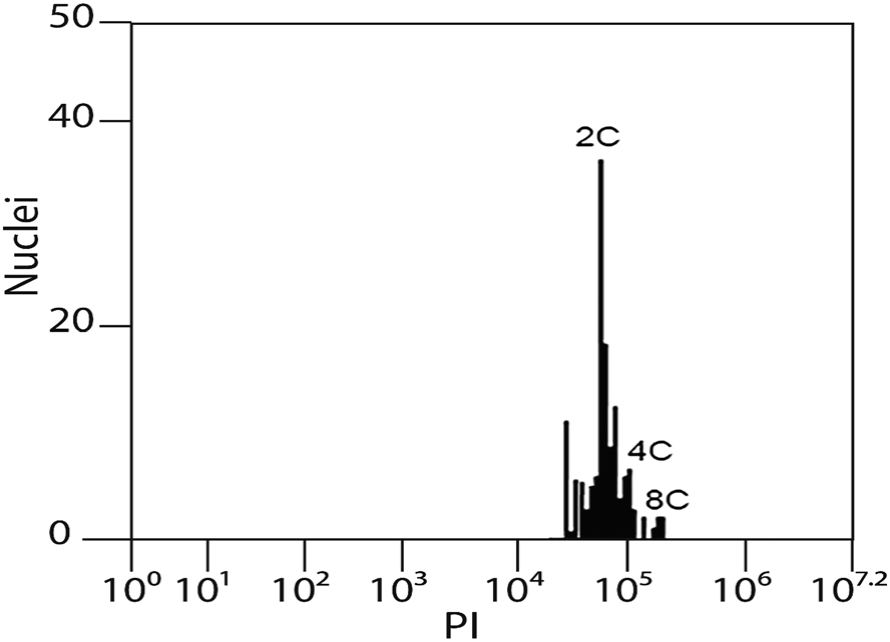
Representative flow cytometry histogram of *L. sativa* root apical meristem cells after 48 h of EOs exposition. Peak 2C represents diploid nuclei of *L. sativa* (2n = 2x = 18 chromosomes; Koopman et al., 1993) in G0/G1 of the cell cycle. Peak 4C represents diploid nuclei in G2/M and autotetraploid nuclei in G0/G1. Peak 8C represents autotetraploid nuclei in G2/M. Cell proliferation was the same of the controls (water and dichloromethane) in all EOs concentrations.

Based on the mean number of G_2_/M nuclei (4C and 8C), the root apical meristem cells showed the same proliferation rate among all treatments. Thus, the EOs of the autotetraploid strains A3 and B1 and diploid 1 from 750 to 3000 ppm were not statistically different from the positive or negative controls (Fig. 3). In cytotoxicity and genotoxicity assays, no chromosome alterations, nuclear disorders, or micronuclei formation were observed. The cells presented well-preserved nuclei and normal mitotic phases even at EOs highest concentration (3000 ppm).

## Discussion

### Ploidy level and chemical composition of EOs

Autotetraploidy promoted chemical diversity in *E. benthamii* EOs, as proved by the occurrence of monoterpenes such as 1,8-cineol, limonene, α-terpineol, and α-terpinyl acetate, which not found previously in *E. benthamii* diploid germplasms. Qualitative differences in EOs were also described in autotetraploid *Trachyspermum ammi* L., in which the EOs extracted from diploid accessions did not present α-terpineol^37^. In *C. limon* EOs, autotetraploids produced β-bisabolene, but this compound was absent in diploid EOs^14^. The changes in EO composition are possibly associated with new gene expression profiles and enzyme activities, which modulate the biosynthesis of secondary metabolites^38^. In this study, we also reported a quantitative change in the α-pinene content of autotetraploids A and B EOs compared with their diploid relatives. Bhuvaneswari et al.^14^ also identified these changes in *C. limon* autotetraploids. The authors attributed the changes to an upregulation of the limonene synthase gene, which led to an increase of 22.23% in limonene content.

The ratio between monoterpenes and sesquiterpenes was also altered in EOs of *E. benthamii* autotetraploids, as they did not contain aromadendrene and viridiflorol sesquiterpenes. The alteration in terpenes derived from secondary metabolism has been described as an euploidy effect, indicating that the increase in ploidy level can modify pathways differently^38^. Considering that *E. benthamii* is predominantly allogamous*,* the differences in EOs compositions among the autotetraploid germplasms indicate that the genotype also contributed to the chemical diversity. Furthermore, there are 113 genes related to terpene synthases in the *E. grandis* genome^13^, and the autotetraploidy increases the number of gene copies per locus.

**Figure 4:**
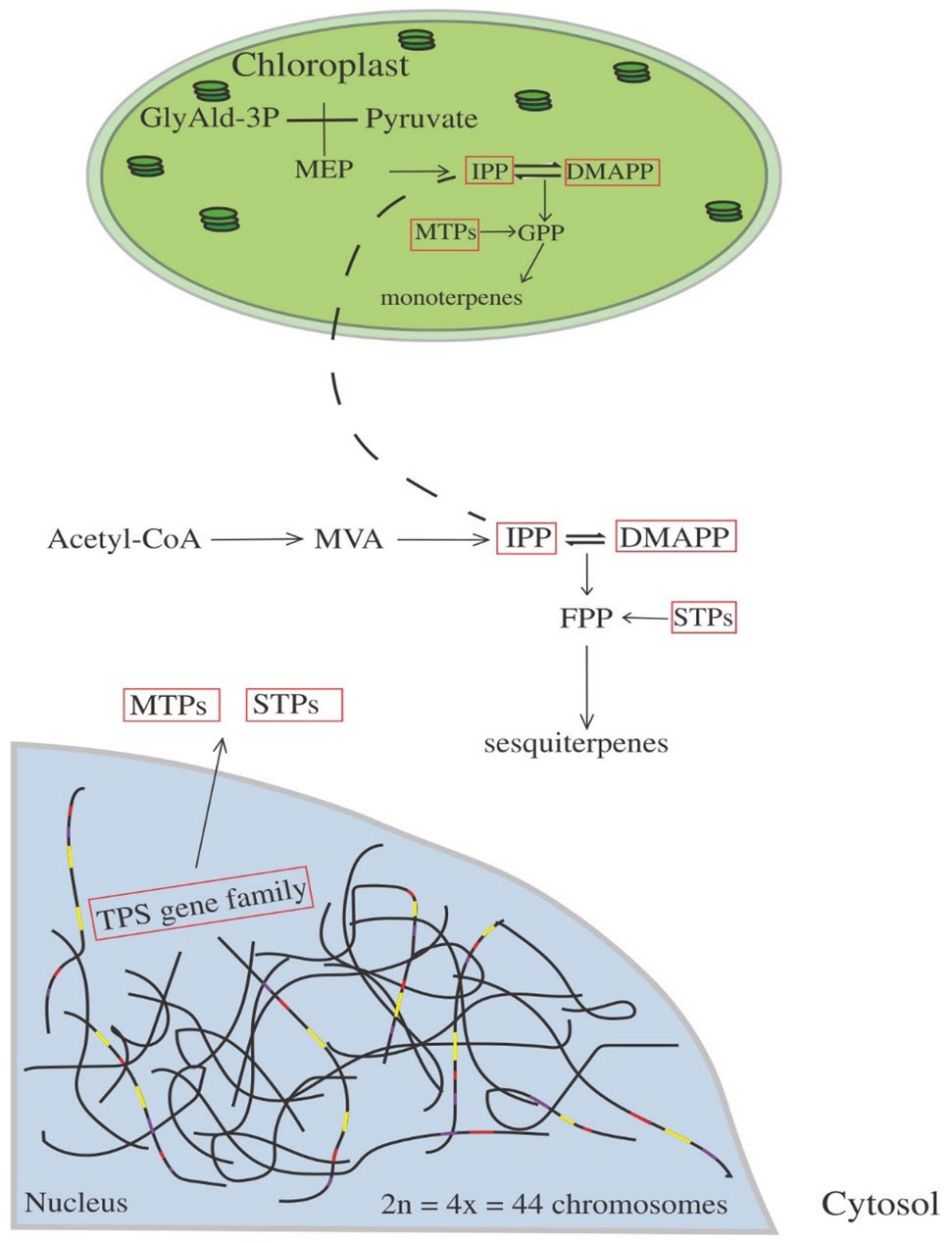
Hypothetical scheme of the terpene pathway in *E. benthamii* autotetraploids. Solid arrows indicated multiple enzymatic and transport steps. Red boxes indicate the role of chromosome set doubling in terpene biosynthesis. Long dashes represent the possible crosstalk between cytosol and chloroplast of the precursor IPP. MTPs are imported to chloroplast and use the MEP products (IPP and GPP) to form the major monoterpenes. STPs use the MVA products (IPP and FPP) to create sesquiterpenes in cytosol. Abberviations: TPS, terpene synthase gene family; MTPs, monoterpene synthases; STPs, sesquiterpene synthases; MVA, mevalonic acid; IPP, isopentenyl diphosphate; DMAPP, dimethilallyl diphosphate; FPP, farnesyl diphosphate; GPP, geranyl diphosphate; MEP, methylerythritol 4-phosphate; GlyAld-3P, glyceraldehyde 3-phosphate.

Hence, autotetraploidy may alter the chemical composition and ratio of mono- and sesquiterpenes differently, according to the pathway explored (Fig. 4). Potentially, CSD would double the number of all the TPS gene family in the nucleus, and consequently, a new gene expression pattern would be set. As antimitotic agents used for CSD only act as spindle inhibitors^39^, the dynamics of gene expression and enzyme activity are related to the doubled nuclear genome (chromosome set). Moreover, monoterpenes predominantly originate from the MEP pathway in chloroplasts, whereas sesquiterpenes, in the cytosol and peroxisomes^40^. So far, the CSD effect in *E. benthamii* supported the MEP pathway, possibly producing more monoterpene synthases that were imported to the chloroplast (Fig. 4). Additionally, geranyl diphosphate synthases compete for the precursor IPP and DMAPP to produce different monoterpenes in chloroplasts^41,42^. Thus, the autotetraploid condition could enhance the precursor availability of IPP and its isomer, DMAPP, as well as geranyl diphosphate synthases in chloroplasts. This would possibly explain the dominance of monoterpene compounds in EOs (Fig. 4).

Therefore, it is possible that autotetraploids have an overdominance effect over diploids, leading to diverse phenotypic changes^16^. The presence of 1,8-cineole, limonene, and α-terpineol has been reported for diploid *E. benthamii* in different Brazilian locations^10,43^. In the present study, these compounds did not occur in diploid germplasms, and the trees were randomly planted in the same field. It is possible that the diploid germplasms did not produce 1,8-cineole, limonene, and α-terpineol because of the quantitative effect of genes that regulate essential oil concentrations. In fact, many quantitative trait *loci* are associated with terpene production *of E. nitens* and *E. globulus*^42,44^, some with larger and others with smaller regulatory effects^45^, which could explain the production of terpenes in different proportions when the genome is doubled.

### Larvicidal activity of diploid and autotetraploid EOs

Diploid EOs that showed high toxicity had a higher percentage of aromadendrene and viridiflorol, indicating that these sesquiterpenes may have a crucial role in larvicidal activity against *A. aegypti*. Lucia et al.^46^ studied 12 *Eucalyptus* species and found a correlation between EOs yield and 1,8-cineole, the higher the percentage of 1,8-cineole the lower the mortality rates of *A. aegypti* larvae. These results could explain the lower toxicity of *E. benthamii* autotetraploid EOs, as they have 1,8-cineole as major compounds and only monoterpenes in its composition. Organophosphate-based insecticides such as temephos are efficient at eradicating *A. aegypti* larvae in concentrations lower than 1 ppm, but these insecticides frequently affect non-target organisms and also lead to larval insecticide-resistance^24^. The chemical diversity of EOs provides a natural barrier against insect resistance due to its chemical complexity^7^, and the diploid EOs of *E. benthamii* can be used as natural biomolecules.

In addition to the lower toxicity, chemical variability presented by autotetraploid EOs may show advantages over their diploid counterparts. Hantao et al.^47^ identified 1,8-cineole and α-terpinyl-acetate as biomarkers for myrtle rust resistance in hybrids of *E. grandis* × *E. urophylla.* Moreover, EOs are involved in plant defense against herbivory, pathogens, and environmental interactions^13^. So far, autotetraploid trees could be used for *Eucalyptus* improvement aimed at plant defense against myrtle rust disease.

### EOs post-emergence activity in *L. sativa*

Considering the phytotoxicity bioassay, there was no influence of EO exposure in diploid, autotetraploids A and B regarding percentage of germination and GSI. In RG, autotetraploids A and B showed an increase in growth (> 5 mm) compared with water controls. Batish et al.^48^*,* using 130 ppm of *C. citriodora* EO*,* reported a root elongation effect in *Amaranthus viridis* and *Echinochloa crus-galli*, revealing that EOs in *Corymbia* and *Eucalyptus* can cause this effect in different plant roots.

In general, EOs from diploid and autotetraploid *E. benthamii* had an inhibitory effect on SG. However, autotetraploids B1 and B3, and diploid 3 had the same effect as the positive control, glyphosate, being statistically similar. The major compounds, α-pinene, found in all germplasms, and 1,8-cineole (autotetraploid germplasms) are also known to inhibit SG in *L. sativa* plantlets under *E. grandis* EOs^33^. As EOs are complex substances, their phytotoxic activity is possibly related to synergism between monoterpenes^49^. Moreover, the non-effect on RG and the remarkable effect on SG indicate that *E. benthamii* EOs act as a post-emergence weed controller, reducing plantlet development, causing a reduction of up to 6.2 mm in the SG of the model plant. Batish et al.^50^ studied the effect of *C. citriodora* EOs, and the authors demonstrated the inhibition of SG, caused by the reduction in chlorophyll content, and the severe inhibition of photosynthesis and respiration. In addition, cell electrolyte activity under *C. citriodora* EOs was enhanced, which possibly altered membrane permeability, leading to oxidative stress^50^.

### Cytogenotoxicity: DNA damage and mitotic agents

Phytotoxicity bioassays generally show the allelopathic potential of EOs over seed germination and EOs influence on plantlet growth and development^51^, Moreover, such bioassays can indicate changes in DNA^52^. In the present study, we evaluated cell proliferation in the roots of *L. sativa* plants treated with control and EO solutions. No significant differences were found in G0/G1 or G2/M phases. These results indicate that the tested EOs are not mitotic promoters that would favor cell division. Moreover, the diploid and autotetraploid EOs of *E. benthamii* did not promote any chromosome alteration or micronuclei formation, which would cause genomic damage. Clastogenic and aneugenic agents cause DNA breaks and dysfunctional chromosome segregation^53^, reducing the mitotic index and, consequently, reducing RG^54^. As those agents were not identified in *L. sativa* roots, *E. benthamii* EOs only showed phytotoxic activity at the initial stages of SG development.

## Conclusions

CSD has been applied in forest species to attend breeding programs. Because trees have long cycle times, it is urgent to describe the effects of CSD in early development. Autotetraploids of *E. benthamii* produced 1,8-cineole, limonene, α-terpinyl-acetate, and α-terpineol, not previously identified in diploid EOs. These chemical changes were related to the increase in ploidy levels and its many “omics” effects. Hence, the genotype differences also contributed to the chemical diversity (autotetraploid A produced α-terpineol, while autotetraploid B, limonene and α-terpinyl acetate). Despite the diversification in EO compounds, the autotetraploid condition did not increase the EO yield (autotetraploid B had the same yield of diploid germplasms).

Exploring the context of chemical diversity in bioassays, diploid germplasms were twofold more lethal against *A. aegypti* larvae than autotetraploids. This could be explained by the sesquiterpenes constitution of the diploid EOs, while autotetraploids only produced monoterpenes. In phytotoxicity bioassays, the major effect of EOs was on SG, acting as a post-emergence controller in *L. sativa* and compared to the glyphosate herbicide. Thus, the EOs of diploid and autotetraploid germplasms of *E. benthamii* have an impact only in the aerial parts of plants, because there was neither genotoxic nor antimitotic properties on cell proliferation in root apical meristems.

Therefore, the increase in ploidy level leads to chemical diversity in *Eucalyptus* secondary metabolites, altering the ratio of mono/sesquiterpenes in EOs. Despite the low toxicity of autotetraploid EOs against *A. aegypti* larvae, 1,8-cineole and α-terpinyl-acetate, present in autotetraploids EOs, were linked as biomarkers to myrtle rust disease. Furthermore, phenotypic selection through the autotetraploid germplasms is required to achieve a greater quantity of EO yield and specific major compounds, considering the scope of the breeding program.

## Supplementary Information

Supplementary Table S1.

Supplementary Table S2.

Supplementary Table S3.
