# Supplementary material for: Short-term changes related to autotetraploidy in essential oil composition of Eucalyptus benthamii Maiden & Cambage and its applications in different bioassays
Source: Sci Rep. 2021 Dec 23;11:24408. doi: 10.1038/s41598-021-03916-2 (PMC8702542; doi:10.1038/s41598-021-03916-2)
Supplement: Supplementary file 1 — Supplementary Table S1. [file 41598_2021_3916_MOESM1_ESM.docx]

| **Table S1:** Chemical composition of EOs extracted from leaves of diploid (2x) and autotetraploid (4x) germplasm of *E. benthamii* trees. Yield and chemical compounds were expressed in percentage (%). (1), (2) and (3) were the samples used of each germplasm selected based on yield (lower, intermediate and higher, %), and thereafter used to perform the bioassays. | | | | | | | | | |
| --- | --- | --- | --- | --- | --- | --- | --- | --- | --- |
| Sample | Germplasm | Yield | Aromadendrene | 1,8-cineole | Limonene | viridiflorol | α-pinene | α-terpineol | α-terpynil acetate |
| 1 | 2x (2) | 1,75 | 22,1 | 0 | 0 | 18,74 | 59,16 | 0 | 0 |
| 2 | 2x | 2,236 | 20,86 | 0 | 0 | 20,7 | 58,44 | 0 | 0 |
| 3 | 2x | 1,258 | 20,16 | 0 | 0 | 19,3 | 60,54 | 0 | 0 |
| 4 | 2x | 1,086 | 16,11 | 0 | 0 | 18,48 | 65,41 | 0 | 0 |
| 5 | 2x | 1,532 | 17,95 | 0 | 0 | 17,7 | 64,35 | 0 | 0 |
| 6 | 2x (1) | 1,162 | 16,92 | 0 | 0 | 19,78 | 63,3 | 0 | 0 |
| 7 | 2x | 1,524 | 18,3 | 0 | 0 | 21,02 | 60,68 | 0 | 0 |
| 8 | 2x | 1,184 | 21,69 | 0 | 0 | 21,88 | 56,43 | 0 | 0 |
| 9 | 2x (3) | 2,3 | 22,61 | 0 | 0 | 21,83 | 55,56 | 0 | 0 |
| 10 | 4x A | 2,03 | 0 | 59,66 | 0 | 0 | 28,58 | 11,76 | 0 |
| 11 | 4x A (1) | 1,792 | 0 | 58,72 | 0 | 0 | 29,21 | 12,07 | 0 |
| 12 | 4x A | 1,998 | 0 | 54,65 | 0 | 0 | 32,6 | 12,75 | 0 |
| 13 | 4x A | 1,718 | 0 | 60,2 | 0 | 0 | 25,73 | 14,07 | 0 |
| 14 | 4x A | 2,588 | 0 | 55,96 | 0 | 0 | 31,73 | 12,31 | 0 |
| 15 | 4x A | 2,056 | 0 | 55,48 | 0 | 0 | 32,13 | 12,39 | 0 |
| 16 | 4x A | 3,766 | 0 | 56,52 | 0 | 0 | 30,15 | 13,33 | 0 |
| 17 | 4x A (3) | 7,214 | 0 | 55,93 | 0 | 0 | 31,14 | 12,93 | 0 |
| 18 | 4x A (2) | 4,31 | 0 | 54,32 | 0 | 0 | 33,17 | 12,51 | 0 |
| 19 | 4x B | 1,398 | 0 | 53,61 | 5,35 | 0 | 32,4 | 0 | 8,64 |
| 20 | 4x B (2) | 1,732 | 0 | 51,19 | 5,47 | 0 | 35,49 | 0 | 7,85 |
| 21 | 4x B | 0,912 | 0 | 52,48 | 5,41 | 0 | 34,09 | 0 | 8,02 |
| 22 | 4x B | 0,86 | 0 | 52,32 | 5,2 | 0 | 34,62 | 0 | 7,86 |
| 23 | 4x B | 1,282 | 0 | 51,83 | 5,47 | 0 | 34,5 | 0 | 8,2 |
| 24 | 4x B | 1,306 | 0 | 53,57 | 5,59 | 0 | 32,56 | 0 | 8,28 |
| 25 | 4x B (3) | 1,986 | 0 | 49,78 | 5,1 | 0 | 31,45 | 0 | 8,62 |
| 26 | 4x B (1) | 0,648 | 0 | 52,1 | 5,14 | 0 | 34,13 | 0 | 8,63 |
| 27 | 4x B | 0,95 | 0 | 56,52 | 0 | 0 | 33,79 | 0 | 9,69 |
