# Supplementary material for: Short-term changes related to autotetraploidy in essential oil composition of Eucalyptus benthamii Maiden & Cambage and its applications in different bioassays
Source: Sci Rep. 2021 Dec 23;11:24408. doi: 10.1038/s41598-021-03916-2 (PMC8702542; doi:10.1038/s41598-021-03916-2)
Supplement: Supplementary file 2 — Supplementary Table S2. [file 41598_2021_3916_MOESM2_ESM.docx]

Table S2. Identification of essential oil compounds.



| n | Compound^a^ | RI_cal_^b^ | RI_tab_^c^ |
| --- | --- | --- | --- |
| 1 | α-pinene | 936 | 932 |
| 2 | limonene | 1030 | 1024 |
| 3 | eucalyptol | 1033 | 1033 |
| 4 | trans-β-ocimene | 1043 | 1044 |
| 5 | α-terpineol | 1191 | 1186 |
| 6 | α-terpinyl acetate | 1352 | 1346 |
| 7 | aromadendrene | 1438 | 1439 |
| 8 | viridiflorol | 1584 | 1592 |

^a^Major compounds listed in elution order using Rtx^®^-5MS column. ^b^Retention index calculated using saturated n-alkanes (C_7_-C_40_) sample data. ^c^Retention index database (ADAMS, 2007; EL-SAYED, 2016; NIST, 2011).
