# Supplementary material for: Short-term changes related to autotetraploidy in essential oil composition of Eucalyptus benthamii Maiden & Cambage and its applications in different bioassays
Source: Sci Rep. 2021 Dec 23;11:24408. doi: 10.1038/s41598-021-03916-2 (PMC8702542; doi:10.1038/s41598-021-03916-2)
Supplement: Supplementary file 3 — Supplementary Table S3. [file 41598_2021_3916_MOESM3_ESM.docx]

| **Table S3:** Death (%) of *A. aegypti* larvae after 24h of exposition to diploid (2x) and autotetraploid (4x) EOs. The numbers (1), (2) and (3) refers to the yielding classified as lower, intermediate and higher EO yield (%). | | | | | | | | | |
| --- | --- | --- | --- | --- | --- | --- | --- | --- | --- |
| ppm | 2x (1) | 2x (2) | 2x (3) | 4x A(1) | 4x A(2) | 4x A(3) | 4x B(1) | 4x B(2) | 4x B(3) |
| 50 | 0 | 0,8 | 4 | 0 | 0 | 4 | 0 | 0,8 | 0,8 |
| 75 | 0,8 | 4,8 | 39,2 | 0 | 0 | 4,8 | 0,8 | 0,8 | 1,6 |
| 100 | 3,2 | 92,8 | 79,2 | 0 | 0 | 5,6 | 0 | 3,2 | 2,4 |
| 125 | 2,4 | 96,8 | 84 | 0,8 | 0,8 | 9,6 | 4 | 4 | 5,6 |
| 150 | 14,4 | 100 | 92,8 | 9,6 | 8 | 15,2 | 61,6 | 17,6 | 7,2 |
| 175 | 80 | 100 | 96,8 | 41,6 | 39,2 | 40 | 70,4 | 44 | 9,6 |
| 200 | 97,6 | 100 | 100 | 81,6 | 76 | 41,6 | 75,2 | 91,2 | 16 |
